# Identifying priorities for quality improvement at an emergency Department in Ghana

**DOI:** 10.1186/s12873-017-0139-0

**Published:** 2017-08-30

**Authors:** Annelies DeWulf, Elom H. Otchi, Sari Soghoian

**Affiliations:** 1 Department of Emergency Medicine, Louisiana State University Health Sciences Center, New Orleans, USA; 2 Public Health Unit, Korle Bu Teaching Hospital, Accra, Ghana; 3 Department of Emergency Medicine, Korle Bu Teaching Hospital, Accra, Ghana; 4 New York University School of Medicine, New York, USA

**Keywords:** Access to healthcare, Global health, Quality improvement

## Abstract

**Background:**

Healthcare quality improvement (QI) is a global priority, and understanding the perspectives of frontline healthcare workers can help guide sustainable and meaningful change. We report a qualitative investigation of emergency department (ED) staff priorities for QI at a tertiary care hospital in Ghana. The aims of the study were to educate staff about the World Health Organization’s (WHO) definition of quality in healthcare, and to identify an initial focus for building a departmental QI program.

**Methods:**

Semi-structured interviews were conducted with ED staff using open-ended questions to probe their understanding and valuation of the six dimensions of quality defined by the WHO. Participants were then asked to rank the dimensions in order of importance for QI. Qualitative responses were thematically analyzed, and ordinal rank-order was determined for quantitative data regarding QI priorities.

**Results:**

Twenty (20) members of staff of different cadres participated, including ED physicians, nurses, orderlies, a security officer, and an accountant. A majority of participants (61%) ranked access to emergency healthcare as high priority for QI. Two recurrent themes - financial accessibility and hospital bed availability - accounted for the majority of discussions, each linked to all the dimensions of healthcare quality.

**Conclusions:**

ED staff related all of the WHO quality dimensions to their work, and prioritized access to emergency care as the most important area for improvement. Participants expressed a high degree of motivation to improve healthcare quality, and the study helped with the development of a departmental QI program focused on the broad topic of access to ED services.

**Electronic supplementary material:**

The online version of this article (10.1186/s12873-017-0139-0) contains supplementary material, which is available to authorized users.

## Background

In the past decade, quality improvement (QI) has emerged as a priority for health systems worldwide [1]. An increasing body of evidence suggests that strengthening capacity for emergency care is one cost-effective means to reduce preventable causes of morbidity and mortality and improve healthcare quality [2]. The World Health Organization (WHO) has argued that expanding and integrating emergency services into existing primary care and public health systems is a priority for global healthcare service improvement [3, 4].

However, despite the growing literature on successful models of healthcare QI, relatively little is known about how to implement successful quality improvement programs outside of a small number of high-income countries [5, 6]. Noting the need for a more robust evidence base for QI globally, the International Federation of Emergency Medicine made an urgent call for more research to build the evidence base for quality indicators and QI strategies in low and middle income countries (LMICs) [7].

The present study investigated priorities for QI at a tertiary care hospital emergency department (ED) in Ghana. At the time it was developed, hospital management had tasked departments to suggest locally relevant, specialty-specific QI metrics, with associated targets for improvement. Since staff ownership of and investment in the QI process was critical for success, the study focused on members of staff in the ED. The aims were two-fold: (1) to educate ED staff about the concept of quality in healthcare, and (2) to identify an initial focus for a staff-driven QI program.

## Methods

The study was a qualitative cross-sectional survey using semi-structured interviews with ED staff of different professional backgrounds to collect data about their concepts of and priorities for quality improvement. The study site was the non-trauma emergency unit of a large urban teaching hospital in Ghana. At the time of the study, this 54-bed unit provided service to an annual average of 8000 adolescents and adult patients aged 13 years and above with a variety of medical and surgical disease conditions.

For the purpose of this analysis we used the WHO 2016 working definition of quality, which is based on the six dimensions of efficacy, efficiency, accessibility, acceptability/patient centeredness, equitability, and safety [1]. We investigated two questions of interest:

1. How do ED staff members relate the WHO dimensions of quality to their own work experience?
2. What is the highest priority target for departmental QI at this time?

A structured interview guide was initially developed by the study team, and finalized with inputs from ED staff. The guide included brief statements describing each of the six dimensions of quality as defined by the WHO, along with a series of open-ended questions about the meaning and relative importance of each dimension. Participants were asked how they would define quality of care, and how they would describe the strengths and weaknesses of their current practice in each dimension. Finally, the participants were asked to rank the dimensions of quality in order of importance. A copy of the interview guide is included as an additional file [see Additional file 1. Study Questionnaire].

Study participants were a convenience sample of 20 ED staff stratified by cadre, including one security guard, one accountant, three doctors, five orderlies, and ten nurses. Participants were 55% women, and 45% men. Staff were initially approached by a study author not employed by the hospital (ADW) based on relationships formed during an observational period in the ED. They were recruited if they expressed interest in contributing or were recommended by peers as opinion leaders within the department. Subsequently participants volunteered based on word of mouth.

Inclusion criteria were voluntary participation and one year or more experience working in the ED. Staff meeting these criteria numbered 240 individuals, including six security guards, eight accountants, eight doctors, 13 orderlies, and 202 nurses. All cadres were eligible for participation, and an effort was made to recruit at least ten nurses and one member of each additional cadre based on their relative numbers within the total staff mix. Staff who were not fluent in English were excluded.

Interviews lasted up to one hour and were conducted confidentially over a two-week period in August 2013 by ADW, who shielded the participants’ identities from the other study authors, who are both hospital staff. Participants each signed a consent form and gave permission to audio-record the interview. The consent form and all interview questions were read out loud to the participants in English. The study was exempt from review by the State University of New York Institutional Review Board (study number 67854–3).

Data analysis primarily consisted of thematic analysis of de-identified interview transcripts using Dedoose™^.^. Interview transcriptions were analyzed through multiple readings and then selectively coded along thematic lines, including the six WHO dimensions of quality, opportunities for improvement, and areas of strength. The coding scheme was reviewed and revised by the study team after initial coding to ensure goodness-of-fit between the data and coding scheme. Common themes were determined by comparing the number of statements made in reference to each. Rank order data was analyzed for means, modes, and grouped into categories of high (rank order 1 or 2), medium (rank order 3 or 4) and low (rank order 5 or 6) priority.

## Results

Responses from eighteen (18) participants were included in the rank order analysis. There was a 90% response rate. Interviews with one nurse and one orderly were excluded from this exercise because they either did not understand the questions or did not understand the task (and ranked all the dimensions as first priority). Overall, accessibility was the highest ranked quality dimension (61%) followed by efficacy, safety, and acceptability. Less than 40% of participants ranked any other dimension as high priority (see Table 1). Equity was ranked lowest; 11% of participants ranked it high priority, whereas 61% ranked it as low priority.

**Table 1:** Rank order of QI dimensions

Dimension Of Quality in Health Care	Mode of rank order # responses (%)	Mean rank order	High Priority: 1 or 2 # responses (%)	Medium Priority: 3 or 4 # responses (%)	Low Priority: 5 or 6 # responses (%)
Accessible	1 (50%)	2.61	11 (61%)	2 (11%)	5 (28%)
Effective	1 (28%)	3.33	7 (39%)	7 (39%)	4 (22%)
Safe	4 (33%)	3.28	5 (28%)	11 (61%)	2 (11%)
Acceptable	2,5 (each response 28%)	3.78	6 (33%)	3 (17%)	9 (50%)
Efficient	3 (33%)	3.83	3 (17%)	9 (50%)	6 (33%)
Equitable	6 (50%)	4.55	2 (11%)	5 (28%)	11 (61%)

Nineteen (19) interviews were included in the qualitative analysis. One was terminated early and excluded because of challenges with English language communication. All the participants who were included articulated appropriate case examples in reference to the six dimensions of quality when asked to describe strengths and weaknesses of their current practice. Analysis of qualitative data corroborated the importance of accessibility to the participants. Equity was the second most frequently discussed dimension despite its low rank. Overall, the responses to open-ended questions centered on two recurring topics linked to negative effects on healthcare quality: financial accessibility, and inadequate hospital bed capacity.

### Financial accessibility

Financial accessibility was described as an important barrier to healthcare quality across all six dimensions. Participants frequently explored the concept of affordability, noting that patients and their families often lack the financial means to access healthcare services. The inability to pay for transportation to a hospital or for healthcare services such as medications, laboratory investigations, and radiography was often described as a challenge for quality.

*And at times too, you see in Ghana here, we are not living in good conditions. In terms of poverty. You see, if the person is sick…instead of him to go to the hospital, he’ll feel that he does not have the money. So let him try the herbs…which is not the proper way it must go. So before you get to hospital, things have become worse…You see if you’re sick, and you…report quickly to the hospital, you will be treated. But if you stay in the house because of poverty…you be sitting around…to die.*

This participant links avoidance or delay in seeking needed medical care to fear that the cost will be too great a burden. Because of negative effects on health outcomes, delays in obtaining medical care associated with time spent in mobilizing financial resources were linked to impacts on efficacy, and equity in healthcare.

*Many, many, many more poor. Living in poor conditions. Poor areas. In terms of just malaria mosquitos biting a lot of people. And so, the things start from that….A white man…one day he cough…the next day, he will take him to the hospital…To find the cause, quickly, then he clear it away. But poor person, the child will cough, for about two weeks, before they take him to hospital. Ooh, they, the lungs, are totally collapsed…A rich person, after being sick for two days, goes to treatment. But poor person, who has to choose between hospital and food, will wait much longer. So it make, in Ghana here, the quality, I mean, health care is not all that can represent…*

A National Health Insurance Scheme (NHIS) exists in Ghana, which provides low-cost health insurance for basic medical care on an opt-in basis to anyone residing in the country, however most participants noted that often those treated in public hospitals are not registered for this service and gave examples of services not covered under NHIS for which the costs may be prohibitive for poor patients. Examples included Computed Tomography scans, supplemental oxygen, and “name-brand” medications, which were perceived to have a lower risk of being counterfeit as compared to generic medications.

### Hospital bed capacity

Limited bed capacity was the most frequently addressed theme in all the interviews, linked to every dimension of quality. Every participant linked hospital bed capacity to the concept of accessibility, emphasizing its importance in their concepts of quality healthcare.

*So that means that patients don’t get access because of this bed issue…The doctors are there, the nurses are there, but there are no beds…we direct [the patients] to different hospitals, the following day they come back to tell us they didn’t get beds. So they had to go home, and return the next day*

Most participants described the presence or absence of an available ED bed as a prerequisite for healthcare, and therefore a critical determinant of all other dimensions of quality.

*Because last week Friday, there was a case that came, referred from clinic. Came here and there wasn’t a bed. So they run through the traffic, in order to get to another health facility. The patient popped on the way. The patient died. So, here, it’s because the health sector was not accessible to him, or he couldn’t get to the hospital where he would have access to a bed, thus to a doctor…he may die anyway, but this time, it’s because of the omission of accessibility of the health sector.*

*I would say access [here] is not the best at all…because it seems as if everything we do here is based on our bed capacity, which is very poor. And, in the sense that…people know us to be the finest, to be the “last stop”…it means that nobody else can do anything. You are the only one who can save the situation. So if you say, you don’t have space, then, it means nothing can be done.*

These participants describe limited bed capacity as a rate-limiting step in the process of healthcare provision. Since this referral hospital is seen as the most resourced hospital in terms of specialist services and resources, it is also seen as patients’ last recourse for health services. If no beds are available in the ED, the health care sector in general seems unavailable to patients.

Participants also discussed ED and hospital bed availability in relation to healthcare efficiency and efficacy:

*Early morning you have to go round, round, round, fighting, struggling to find a bed for a patient to lie on. Before maybe you get the patient to bed, the patient is dead. Or even the condition is worse, has been made worse because of where the patient was before you had the bed to transfer the patient on.*

Acceptability was also highly linked to the problem of bed capacity. For example, participants described facing negative media attention and community outrage from patients being turned away due to beds being unavailable, as well as hostility from desperate patients and their families as they sought access to hospital services.

*If something is not done as soon as possible to change, it will get to a time, nobody would want to come here. Because they would already presume there is no bed. And [die] when they are at home. So they will not even want to give it a try. Because they know when they get themselves here, we will tell them there is no bed.*

This participant expressed concern that chronic access problems had negatively impacted the social standing of hospital staff within their communities. In addition to perceived negative effects on patient, family, and community perceptions of quality, healthcare inaccessibility from inadequate bed capacity was often cited as a source of frustration, anxiety, and helplessness among staff themselves.

*‘Cause it’s quite unfortunate, somebody’s really sick, he needs an attention, …and we tell the person there’s no bed. And because of that, the person’s not going to get access. It’s quite unfortunate, but, it’s also - for you the health care worker - you have moved from your house to come and save your patients. You come, the place is full. Then the person needs a bed to be treated…Here is the case there is no bed and the person is unconscious, cannot sit in the chair, the bed is not there. What do you do? There is nothing you can do….*

Participants also expressed concerns that inadequate bed capacity limits their ability to deliver safe care. For example, some described using what they perceived to be inadequate infection control practices when cleaning beds in between patient encounters because of pressures to maintain access. The implication is that inadequate access to care also places both patients and staff at higher risk for disease transmission. Others described using broken beds or beds without side-rails to maintain bed numbers, potentially placing patients at risk for injury.

### Suggestions for improvement

The participants in this study expressed being highly motivated to provide the highest quality of care possible within the limitations of their practice environment. Suggestions for QI mainly addressed access to emergency care. Many participants characterized the bed capacity problem as a concrete issue of inadequate space and beds, and suggested that the remedy would be to construct additional buildings and procure additional beds. However a few suggested that process improvements such as decreasing ED and hospital “throughput” time could help remedy the situation. One suggested that initiatives to improve staff training, education, and access to resources at district and regional hospitals and health centers might decrease the number of patients who are referred onward to the over-burdened tertiary care centers in the country.

## Discussion

The aims of this investigation were to educate ED staff in our institution about the concept of quality in healthcare as previously defined by the WHO, and to identify priority targets for a staff-driven QI program. Our results demonstrate that participants assigned the highest degree of importance to accessibility, among all the dimensions, and that improving ED access by increasing bed availability was their highest priority for departmental QI. Importantly, participants in this study related concepts of quality to job satisfaction, expressed a high degree of personal investment in providing quality healthcare, and were generally optimistic about the ED’s ability to conduct a successful departmental QI program.

The study was a useful exercise for developing this program since it helped staff crystallize a broad aim to work toward (improve access). Since it was conducted, the ED has identified and developed interventions to improve patient flow and throughput, including: the introduction of a nurse-led triage system, followed by integration of doctors into the triage decision-making process; adopting a policy of moving patients to inpatient beds when these are available, regardless of ability to settle the ED bill at time of transfer; assigning a bed manager role and actively searching for inpatient beds during each shift; and collecting data to track bed occupancy and bed turnover rates, which were subsequently added to the list of performance measures that hospital departments should report on quarterly.

These process changes do not address the larger, and arguably more important, structural health systems issues underlying ED overcrowding, but do represent a form of staff engagement in creating change. The development and success of these departmental initiatives hinged largely upon the input and buy-in of ED staff members, achieved at least in part through this study. Participative, collaborative approaches to problem solving are widely recommended for successful QI [1, 8]. Previous literature on QI in Sub-Saharan Africa, specifically, suggests that involving frontline staff in the process of selecting quality indicators can help elucidate practical solutions to complex problems [9], and have positive effects on staff morale as well as performance [10]. Our experience in conducting this study, and subsequent QI projects within the department, is that staff input into the development of goals and their investment into the approach towards these goals are essential for successful implementation.

The conceptualization of patient and health systems resource limitations as barriers to quality underscores the need for thoughtful analysis of population- and community-specific values, needs, and health systems supports for QI. Although resource limitations are arguably a universal healthcare challenge, and cost-savings are often an important driver for QI activities, there are tremendous inter- and intra-regional disparities in specific health systems deficits and capacities worldwide. Participants in this study described routine failures to meet basic safety and equipment needs such as access to unbroken furniture, clean water, bed-sheets, and quality-controlled medications. In a Donabedian framework, these are all structural measures. The implication is that staff may be unable to offer the intended level of care, and as a result, standard ED performance indicators focused on more upstream processes and outcomes may need careful consideration and adaptation for meaningful use in this setting [11–13].

Although the results of this study cannot be readily generalized to other settings or hospitals in Ghana, participants’ emphasis on bed capacity resonates with experiences of and common approaches to quality in emergency care globally. ED overcrowding is a major challenge worldwide, with well-documented effects on efficiency, safety, access, and patient outcomes [14, 15]. Interventions to reduce ED overcrowding are a common focus for ED and hospital based QI activities [16–20]. Published literature demonstrates that workflow process changes can improve ED throughput times [18, 21] and are cost effective quality interventions [21]. However, no single recipe for success is apparent [16, 22] and the evidence for this is largely limited to case studies from high-income countries. While some efforts have been made towards defining and addressing hospital access issues in Ghana, more work is needed to explore the opportunities and implementation lessons associated with measures to reduce ED overcrowding and access block here as in other LMICs [23].

### Limitations

This investigation is subject to the limitations of self-reported data, and social desirability and researcher biases may have also influenced the results. Self-selection, or non-response, bias is inherent in the study design since participation was voluntary and solicited by word of mouth. Despite this methodological weakness, the recruitment and sampling method carried the advantage of protecting staff from feeling obligated to participate in sensitive conversations that could be construed as being critical toward departmental and hospital management. Inter-rater reliability for data interpretation was also not assessed.

## Conclusion

We report a qualitative investigation of healthcare workers’ priorities for QI at a tertiary care hospital ED in Ghana. In this survey, we found that ED staff members of all cadres could relate each of the WHO-defined dimensions of quality to specific strengths and weaknesses in their current practice. Participants uniformly expressed a high degree of personal and professional motivation to improve healthcare quality, and the majority ranked accessibility as the highest priority focus for QI within the department.

Financial accessibility and hospital bed availability were also the most common themes that emerged in relation to opportunities for improvement across other quality dimensions. Access to hospital beds in particular, and to the services they permit, was consistently described as a primary condition for all the other dimensions of quality. This feedback suggested that interventions to increase ED access would have positive effects on both staff morale and the quality of emergency care provided to patients, their families, and the community, and has been useful in the initiation of a staff-driven QI program within the department.

## Additional file

Additional file 1: Study Questionnaire. (DOCX 102 kb)

## Abbreviations

ED: Emergency department
LMICs: Low and middle income countries
QI: Quality improvement
WHO: World health organization
