# Supplementary material for: Identifying priorities for quality improvement at an emergency Department in Ghana
Source: BMC Emerg Med. 2017 Aug 30;17:28. doi: 10.1186/s12873-017-0139-0 (PMC5576337; doi:10.1186/s12873-017-0139-0)
Supplement: Additional file 1: — Study Questionnaire. (DOCX 102 kb) [file 12873_2017_139_MOESM1_ESM.docx]

**Additional 1: Study Questionnaire**

- What does quality in health care mean to you?
  - If you were sick, what do you think you would expect from high quality health care?
  - Do you think your answer is influenced by your knowledge of how emergency care works, because you currently work in the SME?
  - If yes, how?
- What elements do you believe are important for quality to exist in health care in general?
  - What elements do you believe are important for quality of care to exist in the SME?
- Which element you just named is most important to you?
  - In what way is it important?
- What elements of quality in health do you think are most important to patients?
  - Why do you think it is/is not different from your previous answer?
- Many people who study “quality in healthcare” talk about certain aspects. For example, the World Health Organization lists a number of aspects related to quality in health care. I’m going to ask you about each of them
  - “Accessible” means that everyone can get the care offered there.
    - How important do you think accessibility is to providing high quality of care in general?
    - How important is it to care here in the SME?
  - “Acceptable” means that the care provided is seen as a positive experience to patients and their communities, without cultural insensitivity or other unintended bad effects.
    - How important do you think acceptability is to providing high quality of care in general?
    - How important is it to providing care in the SME?
  - “Equitable” means that everyone, regardless of income or job, gets the same care as everyone else.
    - How important is providing equitable care to patients in general?
    - How important is it to providing care in the SME?
  - “Safe” care means that there are few risks in receiving medical care, such as medication errors or unsafe procedures.
    - How important is safety to quality of care in general?
    - How important is safety to quality of care in the SME?
  - “Effective” care means that there are better outcomes, better health and healing, with the help of medical care.
    - How important is effectiveness to quality of care in general?
    - How important is effectiveness to quality of care in the SME?
  - Efficient” care means that time and money are not wasted in the delivery of medical care.
    - How important do you think efficiency is in the delivery of high quality medical care?
    - How important do you think efficiency is in the SME?
- Do you think these above terms explain quality?
  - In what way does it/does it not?
- What other things affect quality in health care?
- On this sheet of paper are the World Health Organization quality measures. I am also going to add in your added response. Can you rank these in order of importance to you?
- When you think of the quality of care provided in the SME, what aspects of quality do you think are done best?
  - Can you describe any examples?
  - How, if at all, has this changed since you started working there?
- When you think of quality of care in the SME, what are some areas where there could be improvement?
  - Can you describe any examples?
  - What do you think has caused this to be a problem area?
  - What has been done or could be done to improve this issue?
